# Supporting medical physics resident development through mentored journal peer review experiences

**DOI:** 10.1002/acm2.70387

**Published:** 2025-11-18

**Authors:** Jessica M Fagerstrom

**Affiliations:** ^1^ Department of Radiation Oncology University of Washington Seattle Washington USA

**Keywords:** education, mentorship, peer review, residency, scholarly development

## Abstract

**Background:**

Although medical physics residents frequently engage with academic literature, many have limited exposure to the peer review process from the reviewer's perspective. Mentored peer review offers a structured, accessible opportunity for residents to develop critical appraisal skills, understand scholarly communication workflows, and participate in academic service. However, many faculty mentors and residency programs lack clear guidance on how to support residents through this process. This work outlines how medical physics residency educators can support a mentored peer review experience for medical physics residents.

**Learning objectives for residents include:**

1. Identifying and explaining key components of the peer review process.
2. Demonstrating the ability to draft a structured, ethical peer review.
3. Reflecting on how peer review supports scholarly growth and professional identity formation.

**Discussion:**

This article outlines actionable strategies to help mentors integrate peer review into medical physics residency training. Topics include orienting residents to editorial workflows, modeling professional feedback, emphasizing ethics, and building independence. These strategies are grounded in published literature and professional best practices and aim to cultivate both competent reviewers and reflective, engaged contributors to the medical physics scholarly community.

## 1 INTRODUCTION AND NARRATIVE

Peer review is a cornerstone of scholarly communication. Yet many medical physics residents, even those who have authored papers, enter residency with limited exposure to the peer review process from the reviewer's perspective. Some will have experience responding to reviewer comments on their own work. Others, especially physicists who did not have publishing as a requirement for their graduate work, will be entirely new to manuscript submission and evaluation. Regardless of background, participating in peer review offers trainees a powerful opportunity to deepen their disciplinary knowledge, sharpen their writing and critical thinking skills, and engage with the broader academic community.

Mentored peer review during residency can scaffold the process in a way that emphasizes learning rather than gatekeeping. Work by Aamodt et al.^1^ suggests that formal peer review mentoring not only supports trainee development but improves the quality of submitted reviews, making an important contribution to journals themselves. Moreover, peer review aligns well with Accreditation Council for Graduate Medical Education (ACGME) expectations for scholarly engagement,^2^ offering a high‐impact and relatively low‐barrier activity that complements more time‐intensive forms of research and publication. As Dwyer et al.^3^ note, peer review can be a particularly accessible avenue for fulfilling possible scholarship requirements within graduate medical education. Some major journals, including the *International Journal of Radiation Oncology, Biology, Physics*, have even established dedicated resident peer review training programs, highlighting growing interest in formalizing this developmental experience.^4^ Although strategies described here are presented in the context of academic residencies, the underlying approach may also be feasible in non‐academic programs, as many clinically oriented physicists maintain scholarly involvement from graduate training or professional activities, and can provide mentorship in peer review. Previous work provides guidance for reviewers aiming to develop their skills and contribute meaningfully to the field.^5^ Building on this foundation, the following discussion offers practical strategies for integrating mentored peer review into residency training. The goal of this work is to help faculty mentors and program directors create experiences that are ethically grounded, educationally meaningful, and professionally empowering, for both the trainee and the scientific community they are learning to serve.

## 2 LEARNING OBJECTIVES

The following learning objectives are intended for residents participating in a mentored peer review experience, as described in this work. They can be used and adjusted by faculty mentors or program directors to guide and assess the resident's development throughout the activity.

1. Identify and explain the stages of the academic peer review process, including submission, editorial triage, and reviewer roles, as they apply to medical physics journals, by the end of the mentored activity.
2. Demonstrate the ability to collaboratively draft a professional, structured, and ethically sound peer review for a medical physics manuscript, using journal‐specific reviewer guidelines, within a 4‐to‐6‐week timeline including the specific 2‐week review period.
3. Reflect on one's development as a reviewer by articulating at least two ways the peer review process has influenced critical reading, academic writing, or professional identity formation within 1 week of review submission.

## 3 DISCUSSION

When working with a resident on their first peer review, orient the learner to what you mean by peer review and why exactly it matters in medical physics. Begin by clearly defining peer review and its role in scholarly communication. Peer review is the process of subjecting research to evaluation by experts in the field, with the dual goals of improving manuscript quality and ensuring that published work meets standards of scientific rigor, originality, and ethical conduct.^6^ This function not only helps support the reliability of scientific knowledge but also serves as a formative experience for reviewers, helping them develop critical reading, writing, and reasoning skills.

Also stress to the learner that peer review is not a perfect system. It can be slow, biased, and inconsistently applied, and it is typically performed as unpaid labor. Nonetheless, it remains a key aspect of academic publishing and scientific trust. As Morley and Grammer^7^ note, while peer review may be flawed, the absence of it, or its replacement with a purely unregulated system, could pose harm to scientific discourse and public trust. Participating in peer review helps sustain scholarly communities, fosters reflective critique, and offers valuable insight into how research is shaped, improved, and interpreted before publication.

By orienting trainees to both the value and limitations of peer review, you establish its relevance not just as a publishing checkpoint, but as an active and critical part of professional scholarly life. This discussion describes some practical strategies for residency educators for this process.

### 3.1 Walk through the editorial workflow together

Before reviewing a manuscript, trainees benefit from understanding where their input fits in the broader editorial process. Begin by outlining the big picture of the full trajectory of a manuscript: submission, editorial triage (which may result in immediate desk rejection), assignment to peer reviewers, revision cycles, and final editorial decision. Explaining these stages clarifies how a trainee's comments are used, the weight they carry in decision‐making, and the rationale behind editorial outcomes. It also prepares them for the varied formats of editorial communication, including conditional rejections, major revisions, and the possibility that a manuscript may ultimately be rejected despite favorable reviews.^6^, ^8^, ^9^

Introduce the different types of peer review systems, such as single‐anonymous (where the reviewer knows the author's identity), double‐anonymous (where both identities are concealed, as with *JACMP* and soon to be *Medical Physics*), and, in some journals, open review, so that trainees understand the expectations around transparency and professionalism in each context.^10^ Providing this context reinforces that peer review is not a standalone judgment, but one component of a complex evaluative process.

Grounding trainees in the editorial process early promotes more focused, constructive, and appropriately scoped reviews. It also fosters respect for the roles of editors, authors, and fellow reviewers within the collaborative ecosystem of academic publishing.

### 3.2 Review the anatomy of a good review

Before diving into a specific manuscript or sample review, help trainees understand the structure and components of an effective peer review. A high‐quality review is more than a summary or a list of complaints or required changes. Instead, it is a clear, balanced, and evidence‐informed assessment of a manuscript's strengths and weaknesses. Walk through the general anatomy of a review: how to open with a concise summary of the paper, how to group comments by theme or manuscript section, and how to differentiate between major and minor concerns.

There are several published helpful frameworks available to guide this process. Mohty and Melo^11^ and Seals and Tanaka^12^ recommend a systematic approach to reviewing each section of a manuscript. They suggest using structured prompts to evaluate clarity, rigor, and scientific validity. Banerjee et al.^9^ propose orienting reviewers with guiding questions. Example questions could interrogate if the research is original and meets ethical standards. These types of scaffolds can help new reviewers stay organized and grounded in the purpose of the review.

Encourage trainees to read the journal's instructions to reviewers. Many journals, including *JACMP*, outline what they expect in a review, including tone, format, and review criteria, and they also stipulate what they expect in a submitted manuscript. Next, walk through examples of reviews you have written yourself with identifying details removed as needed. Concrete examples help demystify the tone, structure, and level of detail expected in peer review. Point out how you framed your comments, balanced critique with constructive suggestions, and organized your feedback for clarity. This kind of modeling serves as scaffolding, providing a foundation the trainee can build on when they begin writing their own review. It also opens space for questions and conversation about norms that are often assumed but rarely taught, such as when to comment in the main text versus as a confidential remark to the editor, or how to handle a paper that seems outside one's expertise. Consider sharing something you would do differently in hindsight (e.g., a point you overlooked or phrased too strongly) to demonstrate that reviews are not perfect, and that growth and reflection are part of the process. Modeling humility helps normalize imperfection and encourages residents to review thoughtfully, not flawlessly.

In addition to reading the journal's instructions to reviewers, also encourage residents to review the journal's instructions to authors, and to evaluate the scope and audience of the journal. Understanding how a manuscript aligns with the journal's aims helps tailor feedback to issues most relevant for the editor and readership. Lastly, residents can begin to develop their sense of what constitutes a quality review by discussing and comparing feedback from multiple mentors. A designated primary mentor provides mentor accountability and thorough guidance, while input from additional mentors offers diverse perspectives that enrich reflection and promote a more well‐rounded understanding of the peer review process.

### 3.3 Teach the ethics of peer review and acknowledge limitations of peer review

Before participating in peer review, residents should be explicitly taught the ethical expectations of the process. Peer review is a professional responsibility built on trust, integrity, and confidentiality. Trainees must understand that the content of the manuscript and all associated materials are strictly confidential. These should not be discussed outside the review process. This includes prohibiting the use of generative AI tools to write or summarize submitted manuscripts, since such practices violate confidentiality and risk the introduction of factual errors or biased output. Reviewers must avoid both real and apparent conflicts of interest, as even the perception of bias can undermine the integrity of the review process. This includes relationships with authors, institutions, or competing research groups. They should also evaluate whether they have the appropriate expertise and the time to complete a fair and thorough review.

As Rockwell^13^ notes, ethical reviewers are obligated to flag concerns such as plagiarism, duplicate publication, or previously published work that lacks appropriate attribution. Trainees should understand that self‐plagiarism (reusing substantial parts of one's own previously published work without citation) is still a form of academic misconduct. Although many journals, including *JACMP*, employ automatic plagiarism detection software to identify textual overlap, these tools are not foolproof. Reviewers play a critical role in recognizing potential ethical concerns, including unacknowledged duplication of study design or results, and should alert editors if such issues arise.

Peer review is an essential but imperfect process. Mentoring residents in how to recognize and mitigate bias strengthens their ability to contribute ethically and thoughtfully to scholarly discourse. Encourage trainees to reflect on their own assumptions and preferences. Are they criticizing the manuscript for what it is, or for what they personally wish it had been? Help them distinguish between methodological rigor and differences in framing, language, or writing style that may reflect cultural or disciplinary norms rather than scientific deficits. Reviewers may unintentionally be influenced by factors such as the authors' institutional affiliation, nationality, gender, or primary language, particularly in journals where author anonymity is partial or absent.^14^ Discuss these dynamics openly and model strategies to reduce their influence, such as focusing comments on the evidence presented, using structured review forms, and re‐reading with an eye toward fairness.

Rockwell^13^ and others have documented how reviewers may privilege familiar methods, dominant voices, positive results, or implicit norms of “good writing” rooted in their own academic training. Teaching trainees to become aware of these biases not only improves the fairness of their reviews but also fosters humility and inclusivity in academic practice. Emphasizing transparency and professionalism helps residents see their role not just as critics, but as stewards of equitable scholarly exchange. It's also important to address language bias directly. Although it's reasonable to note when unclear writing limits the ability to review a manuscript, it is inappropriate to suggest that authors “have it proofread by a native English speaker.” This kind of comment reflects biased assumptions and excludes multilingual scholars.^15^ Instead, reviewers can respectfully suggest that the manuscript be reviewed by someone fluent in professional or academic English, if clarity is a barrier to evaluation. Such feedback focuses on the writing itself, not the writer.

### 3.4 Start with a familiar topic

When mentoring a resident in peer review, it's best to begin with a manuscript that falls squarely within their area of expertise. Choosing a short, accessible paper aligned with the trainee's clinical, research, or academic background allows them to concentrate on evaluating the manuscript's structure, clarity, and argumentation, rather than struggling to decode unfamiliar content or methods (technical notes submitted to *JACMP* may provide some quality options). Encourage trainees (and all reviewers) to decline reviews that fall outside of their scope of expertise to avoid overreach or unhelpful/misinformed feedback.^8^ Chittum and Bryant^16^ emphasize that early peer review experiences should be carefully tailored to the trainee's prior knowledge and domain familiarity, as this improves both engagement and the quality of learning. By starting with a paper that the trainee is well‐equipped to evaluate, you promote thoughtful analysis and set the foundation for successful, independent peer reviewing.

### 3.5 Build toward independence

Peer review is a skill developed through supported practice and gradual autonomy. After an initial orientation, progressively shift responsibility to the trainee, allowing them to take the lead. Ask the trainee to write their review independently before seeing the mentor's version to preserve the opportunity for unfiltered critical thinking and help identify areas where their analysis or framing might differ from that of the mentor. Afterward, compare and discuss your respective reviews together. Treat the discussion like an editorial meeting: What did they catch that you missed? Where might their feedback be too lenient, too harsh, or not actionable?

This progression not only reveals gaps in reasoning or knowledge but also builds the trainee's confidence in their ability to assess and articulate scientific critique. Modeling the editor's perspective during feedback encourages clarity, fairness, and attention to impact. Over time, residents (and eventually, early‐career researchers out of training) can be empowered to conduct solo reviews, with you serving as backup if needed.

This approach aligns with findings from a 15‐week peer review course in which medical students gradually assumed primary responsibility for real manuscript reviews. Students gave positive feedback and many continued to review independently after the course ended, suggesting that progressive responsibility supports lasting engagement in academic scholarship.^17^

### 3.6 Model constructive and professional feedback

Trainees may struggle to find the right tone in peer review, especially when they identify major flaws. Use this opportunity to model how to offer honest, critical feedback in a respectful and constructive manner. Instead of judgmental statements, show how to reframe concerns as improvement opportunities. For example: “This section could be strengthened by clarifying the rationale for the study design…” Emphasize the importance of specific, actionable suggestions that authors can use to improve their work.^18^ You can also suggest the resident include at least some positive or encouraging observations in their reviews.

Language matters. Help residents learn to advocate for clarity, rigor, or transparency without sounding condescending or dismissive. Critiques should be respectful, focused, and substantive, not nitpicky. Encourage them to adopt a tone of professional dialogue, imagining themselves in conversation with the authors. As the work of Watling et al.^19^ wryly cautions, no one wants to be (or receive a review from) “Reviewer 2,” a stereotype for an excessively negative, vague, or pedantic reviewer who undermines rather than improves a manuscript. As Weaver et al.^20^ highlight, reviewers must balance critique with civility and avoid overstepping their knowledge base. Teaching these norms early helps trainees enter scholarly communities as trusted and responsible contributors.

Lastly, acknowledge the goal and challenge of completing the review in the requested timeframe. Because many journals require reviews within 2 weeks, this timeline may be difficult for residents juggling demanding rotations and clinical responsibilities. Programs and mentors can mitigate this by requesting deadline extensions from the editorial office when needed or accepting reviews during lighter clinical periods to ensure residents can participate meaningfully without undue burden.

### 3.7 Highlight the personal professional academic value of peer review participation

Participating in peer review can sometimes feel like invisible labor, but it also offers substantial benefits for residents’ scholarly and professional growth. Framing review work as more than a check‐box task in residency helps foster intrinsic motivation and reinforces that this is a valuable scholarly activity and not just another residency requirement. Peer review can be defined as a formal competency within residency training, aligning with existing emphases on scholarship and professional identity formation. Presenting peer review in this way positions it as an integral scholarly skill and recognized component of professional development, rather than an optional or extracurricular task. Peer review allows trainees to step into the reviewer's perspective, sharpening their own writing and reading skills by identifying what makes a manuscript succeed or fail. Exposure to common pitfalls in manuscript preparation, ethical considerations, and stylistic expectations enhances their readiness as future authors.

Peer review also serves as an authentic opportunity to build transferable scholarly skills. McNair et al. identified eight such skills that early‐career researchers may develop through the process: critical thinking, journal‐style writing, working to deadlines, ethical expectations, shaping and constructing research, proofreading, planning, and experimental procedure.^21^ Mayer et al. note that this type of engagement provides a more complete picture of the entire process of academic scholarship, compared to journal clubs, which generally only present finished products,^22^ while fostering habits of professional feedback and constructive critique. Involvement in the editorial process may lead to future tangible professional benefits, such as invitations to join editorial boards or serve in associate editor roles,^20^ and some journals and affiliated professional societies provide other practical benefits for reviewers such as discounts on publication fees or continuing education credits.

### 3.8 Encourage reflection following review submission

Structured reflection after submitting a peer review can deepen and reinforce learning. Use this opportunity to foster metacognitive awareness^23^ by asking the resident questions such as: “What aspects of the manuscript did you find most challenging to assess?”, “What did you learn about research design, writing, or argumentation?”, and “How might this process change how you read or write a manuscript in the future?” These reflective prompts help trainees internalize lessons from the review process and connect them to their personal scholarly development.

Previous work has shown that residents mentored in peer review, when invited to reflect on their ability to understand and apply scientific literature, reported higher self‐perception scores than those who did not participate in a structured peer review program.^24^, ^25^ This reflective practice can be brief but powerful. Even a short conversation or written prompt can elicit insight and growth.

A meaningful peer review experience doesn't end with clicking “submit.” Maintaining post‐review engagement helps residents build a fuller understanding of the publication process and reinforces the professional norms of scholarly contribution, as well as promotes a sense of belonging in the academic community and formation of professional identity.^26^ Ask follow‐up questions such as, “What did you learn from the editor's letter?” These conversations help contextualize the review's impact and normalize variability in editorial decisions. Consider inviting the resident to co‐review another manuscript in the future; this continuity allows for growth, increasing independence, and the opportunity to reinforce constructive habits. Make a point to thank the resident for their work, as recognition helps validate their contribution and encourages future involvement. Ongoing mentoring also helps residents stay connected to the academic community.

## 4 CONCLUSION

Peer review offers a unique opportunity for residents to engage in scholarly service while building essential academic skills. When structured intentionally and supported through mentorship, it becomes more than an isolated task to fulfill during residency; it becomes a formative professional experience. The tips presented here offer practical strategies to guide mentors in scaffolding this process, cultivating not only competent reviewers but also reflective, engaged members of the academic community. As medical education continues to emphasize scholarship and professional identity formation, peer review provides a meaningful and accessible pathway to support these learning goals.

## CONFLICTS OF INTEREST STATEMENT

The authors declare no conflicts of interest.
